# Autochthonous Transmission of *Trypanosoma Cruzi* in Southern California

**DOI:** 10.1093/ofid/ofw227

**Published:** 2016-12-20

**Authors:** Salvador Hernandez, Carmen A. Flores, Gracia M. Viana, Daniel R. Sanchez, Mahmoud I. Traina, Sheba K. Meymandi

**Affiliations:** Center of Excellence for Chagas Disease, Olive View-UCLA Medical Center, Sylmar, California

**Keywords:** autochthonous, California, chagas disease, transmission, *Trypanosoma cruzi*

## Abstract

*Trypanosoma cruzi* usually infects humans via triatomine insects in Latin America. Vector-borne transmission in the United States is exceedingly rare. We describe (1) the first case of probable autochthonous transmission reported in California in more than 30 years and (2) the first ever reported case in the greater Los Angeles area.

Chagas disease (CD), caused by the protozoan parasite *Trypanosoma cruzi*, affects approximately 6–7 million people in Mexico, Central, and South America where it is endemic. The acute phase, lasting 4–8 weeks, is typically asymptomatic or characterized by a self-limited febrile illness. However, the chronic phase may progress to irreversible heart damage in 20%–30% of individuals [1]. Although other modes of human transmission exist, the most important route is through the triatomine insect [2]. During this process, the insect takes a blood meal and defecates shortly thereafter, passing *T cruzi* directly into the wound. Mammalian hosts such as rodents and raccoons are infected in the sylvatic cycle, and in some endemic countries cats and dogs contribute to transmission in the domestic cycle. Living conditions play an important role in disease transmission, with the crevices of adobe houses and thatched roofs being ideal for triatomine infestation in many areas [3, 4].

Although *T cruzi* can be found in triatomines throughout the southern states, CD is not considered endemic to the United States. Reasons for this include improved housing conditions in the US, the predominant sylvatic cycle in the US, which minimizes exposure, and the observation that triatomine species in the US, in most studies, take longer to defecate after feeding; a fact that minimizes the chances of wound infection [4]. Autochthonous transmission has been reported in the US, however, with the first case being reported in Texas in 1955 [5]. It was not until 1982 when the first case of vector-borne transmission of CD was reported in California [6]. There were 7 total cases of autochthonous transmission in the US reported in the medical literature as of 2011 [4]. However, the screening of US blood donors since 2007 led to the discovery of 16 cases of vector-borne transmission in 2012 [7] and another 5 cases of vector-borne transmission in southeastern Texas in 2015 [8]. We present a case of CD that was most likely acquired while the patient was living in California. To the best of our knowledge, this represents the first reported case in California in more than 30 years and the first ever reported case in the greater Los Angeles area.

## METHODS

All clinical care was carried out under the auspices of the Institutional Review Board of the Olive-View UCLA Education and Research Institute, and informed written consent was obtained before treatment. This case was identified during a local blood drive, in which the blood donor had routine CD screening performed on his blood sample. Testing by the blood center included the US Food and Drug Admistration (FDA)-approved Ortho *T cruzi* enzyme-linked immunosorbent assay (ELISA) (Ortho Clinical Diagnostics Inc., Raritan, NJ) as well as the non-FDA-approved radioimmunoprecipitation assay (RIPA) (Quest Diagnostics, Madison, NJ). The Ortho ELISA screens for parasite-specific antibodies, and it is considered positive if the signal-to-cutoff is 1.0 or more. Radioimmunoprecipitation assay also tests for parasite-specific antibodies and is interpreted as either positive or negative, with a positive result confirming reactive antibodies. The Centers for Disease Control and Prevention (CDC), Parasitic Diseases Laboratory, performed additional tests including the Chagatest recombinat version 3.0 ELISA (Wiener Laboratorios, Rosario, Argentina) and the immunofluorescent antibody assay (IFA). The Weiner ELISA is FDA-approved for the diagnosis of CD and usually uses an optical density of 0.33 or more to define positivity. The IFA is generally considered positive with a titer exceeding 1:32. The diagnosis of CD was made based on the combination of a positive Ortho ELISA, RIPA, Weiner ELISA, and IFA, as was done in similar reports of autochthonous transmission in the United States [7, 8].

## CASE REPORT

A 19-year-old healthy white male donated blood in 2009 to a blood drive at a local high school conducted through Providence Health. His blood was screened for *T cruzi* antibodies with the Ortho ELISA, and the positive result was confirmed by RIPA. Upon being notified of these results he first presented to his local pediatrician. This pediatrician contacted the CDC where further testing was performed and results confirmed by ELISA and IFA (Table 1). His evaluation and treatment was then directed by the Center of Excellence for Chagas Disease (CECD) at Olive View-UCLA Medical Center.

**Table 1.** Serologic Test Results

Providence Health (2009)		CDC (2009)		CDC (2012)	
Ortho ELISA	RIPA	Weiner ELISA	IFA	Weiner ELISA	IFA
+	+	+ (1.119)	+ (1:64)	+ (0.920)	-

Abbreviations: CDC, Centers for Disease Control and Prevention; ELISA, enzyme-linked immunosorbent assay; IFA, immunofluorescent antibody assay; RIPA, radioimmunoprecipitation assay.

**Figure 1.**
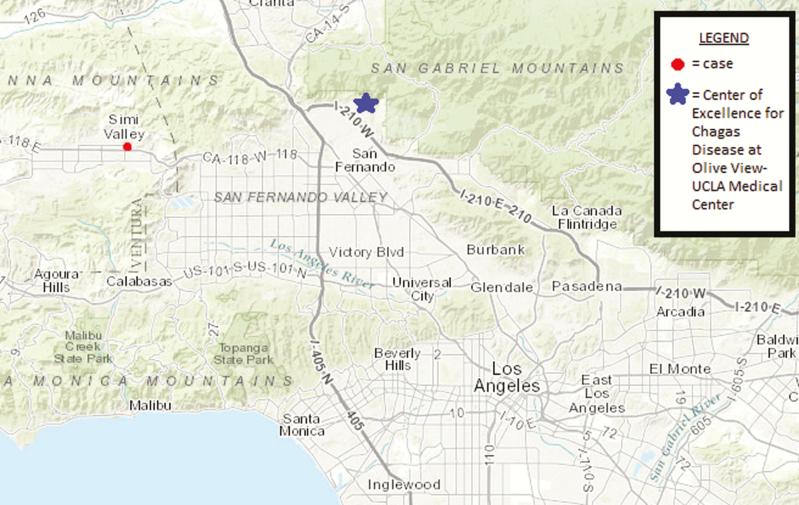
Map of Los Angeles, California, showing the residential location of the case and the Center of Excellence for Chagas disease at Olive View-UCLA Medical Center.

The patient was born and raised in Simi Valley, a suburban city located in Ventura County (Figure 1). He had no symptoms of chest pain, dyspnea, palpitations, or abdominal pain. Past medical history was unremarkable, with no history of blood transfusions or organ transplants. The patient was a full-time student with no occupational exposure to parks or wooded areas. He had no history of significant travel to endemic areas, but he did report frequent outdoor activities including mountain biking in Ventura County and camping throughout southern California in San Diego County, Kern County, and Ventura County. He also visited national parks such as the Grand Canyon, Bryce Canyon, and Zion. His camping activities usually took place in a tent or recreational vehicle. The patient denied having seen the triatomine insect or experiencing unusual bug bites. There was no history of an edematous and erythematous skin reaction (chagoma) or unilateral conjunctivitis associated with an edematous eyelid (Romaña’s sign), which could have indicated a site of parasite entry. His father and mother were born and raised in the US, and their serologic testing was negative for *T cruzi* antibodies by the CDC.

Baseline electrocardiogram, echocardiogram, and Holter monitoring were performed outside the CECD and found to be unremarkable. He was offered antitrypanosomal treatment. Benznidazole was started at a dose of 5.3 mg/kg per day (divided in 2 doses); however, after 9 days of treatment, the patient developed a skin rash and numbness in the fingers that lead to treatment discontinuation. These side effects resolved within 2 weeks of stopping the medication. He subsequently began treatment with nifurtimox at a dose of 300 mg/day (divided in 3 doses), which was gradually titrated up to a maximum dose of 720 mg/day (7.2 mg/kg per day, divided in 3 doses). Side effects of this treatment included anxiety and depression, for which the patient underwent psychotherapy. He was able to successfully complete the entire course of treatment (115 days). During follow-up 3 years later, the Weiner ELISA remained positive; however, the IFA became negative (Table 1).

## DISCUSSION

We describe (1) the first case of probable autochthonous transmission reported in California in more than 30 years and (2) the first ever reported case in the greater Los Angeles area. After extensive history-taking, this patient with CD was found to have no significant travel history outside the US nor any other risk factors that would place him at risk for any other mode of transmission apart from vector-borne transmission. The available evidence suggests that the patient was inoculated with *T cruzi* in the greater Los Angeles area, most likely through contact with the contaminated feces of an infected triatomine insect. More importantly, the patient had exposure to vectors during hunting and camping activities that has been hypothesized as a novel risk factor in the literature [8, 9].

It is worth noting that this case was discovered during a blood drive. Under research protocols, Los Angeles blood banks have detected *T cruzi* antibodies in donor blood since 1994. The most current seroprevalence estimate from 2006 is 1 in 3800 donors, most of which are presumed to be from Latin American immigrants [10]. The American Red Cross began screening all donated blood for *T cruzi* in January 2007, and the case presented here underscores the importance of such screening efforts, not only for the protection of the US blood supply but also for the affected patients themselves. Because the patient presented in this report was in the indeterminate stage of chronic CD, he was a candidate for antitrypanosomal therapy [1].

This report has important limitations. Similar to other case reports, this is a presumed case of autochthonous transmission [7, 8]. Our ability to identify both the mode and site of transmission relies on the self-reported history of the patient. The patient presented here was young, with no extensive medical history to remember and parents who were able to corroborate historical details, which may increase the reliability of his self-reported history. Furthermore, we did not have the resources to further explore this patient’s home with sampling of nearby triatomines and/or mammals as done in previous cases [8, 11].

## CONCLUSIONS

In summary, autochthonous transmission of CD can occur in the US including California [4]. Recent blood donor screening has identified cases of probable vector-borne transmission in multiple southern states [7, 8]. If diagnosed early, antitrypanosomal therapy may improve clinical outcomes [12]. Given the possibility for autochthonous transmission in the US, CD should no longer be considered a disease solely of Latin America.
